# Digital twins in sustainable transition: exploring the role of EU data governance

**DOI:** 10.3389/frma.2024.1303024

**Published:** 2024-03-07

**Authors:** Beatrice Garske, Wilmont Holz, Felix Ekardt

**Affiliations:** ^1^Research Unit Sustainability and Climate Policy, Leipzig, Germany; ^2^Faculty for Environmental and Agricultural Sciences, Rostock University, Rostock, Germany; ^3^Faculty of Law and Interdisciplinary Faculty, Rostock University, Rostock, Germany

**Keywords:** digital twin, Destination Earth, Green Deal, sustainability, data governance, EU data strategy, big data, AI

## Abstract

**Introduction:**

Digital twins can accelerate sustainable development by leveraging big data and artificial intelligence to simulate state, reactions and potential developments of physical systems. In doing so, they can create a comprehensive basis for data-driven policy decisions. One of the purposes of digital twins is to facilitate the implementation of the EU's Green Deal-in line with internationally binding climate and environmental targets. One prerequisite for the success of digital twins is a comprehensive, high-quality database. This requires a suitable legal framework that ensures access to such data.

**Methods:**

Applying a qualitative governance analysis, the following article examines if the EU's strategies and legal acts on data governance are paving the way for digital twin projects which promote sustainability.

**Results:**

Results show important starting points for open and fair data use within the growing field of EU digital law. However, there is still a lot of progress to be made to legally link the use of digital twins with binding sustainability objectives.

## 1 Introduction

**Figure 1:**
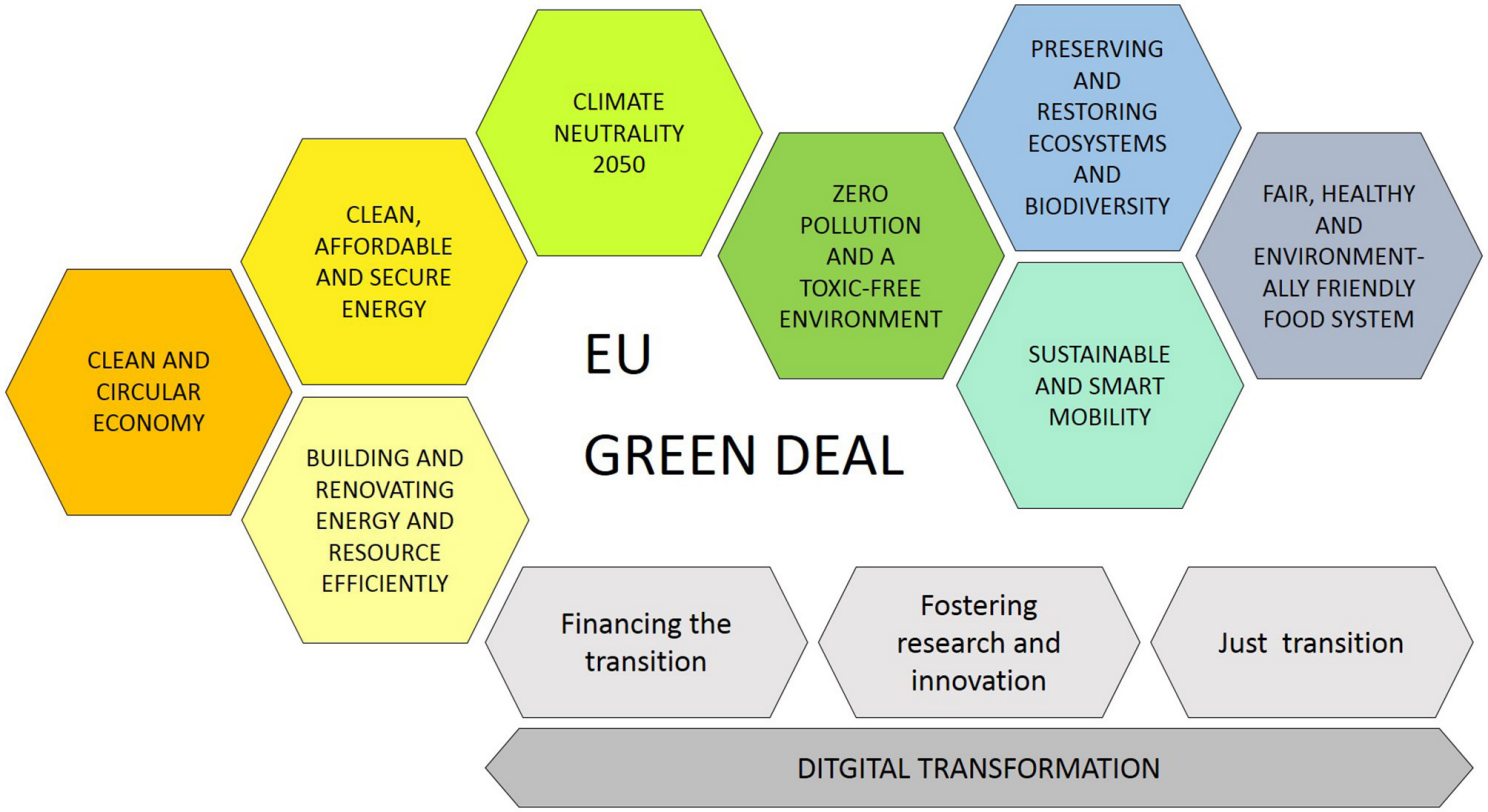
Objectives of the European green deal (own figure based on European Commission, 2019).

The Green Deal (European Commission, 2019) is the EU's attempt to meet the ecological challenges of the twenty first century and to implement the legally binding environmental targets of the Paris Agreement (United Nations, 2015a), i.e., the 1.5°C temperature limit, and of the Convention on Biological Diversity (UNEP, 1992) which aims at halting biodiversity loss. Besides, the policies and laws adopted under the Green Deal should contribute to the achievement of the legally non-binding Sustainable Development Goals (SDGs) (United Nations, 2015b). The key objectives of the European Green Deal are illustrated in Figure 1.

One crucial element of the European Green Deal is an openly accessible and interoperable European dataspace as a central hub for informed decision-making on sustainability issues. Hence, the digital transformation is an important building block of achieving the goals of the Green Deal (European Commission, 2020b; Bauer et al., 2021) and digital and sustainable transition are interlinked in various – also ambivalent – ways (Ekardt, 2022).

Advances in high-performance computing, big data analytics, artificial intelligence (AI) and particularly machine learning (ML) as well as progress in Earth system observation and prediction have enabled increasing precision in digitally representing physical systems (Bauer et al., 2021; Tzachor et al., 2022b; Fissore et al., 2023; Purcell et al., 2023). High quantities of near-real-time data from satellites and sensors are supported by novel observational instruments such as miniaturized satellites, drones, undersea cables and buoys, smart sensor arrays in crop fields and an expanding Internet of Things and – together with physics-based models and ML – allow for highly detailed simulations of real-world objects (Bauer et al., 2021; Li et al., 2023).

Such dynamic, real-time, virtual replicas of physical and biological entities are called digital twins (DTs) (Botín-Sanabria et al., 2022; Kepka et al., 2022; Tzachor et al., 2022b; Fissore et al., 2023; Li et al., 2023; Mehrabi, 2023; Purcell et al., 2023; Ruangpan et al., 2023). They promise deep insights on the functioning of real-world objects and enable predictions about the behavior of the simulated systems over different timescales and under different conditions and constraints (Botín-Sanabria et al., 2022; Tzachor et al., 2022b).

Originally, *in silico* equivalents of real-world objects simulated manufacturing processes in product and process engineering and for space technology (Bauer et al., 2021; Tzachor et al., 2022b). Today, DTs offer substantial modeling potential ranging from molecular to landscape scales, encompassing domains such as meteorology, personalized medicine and public health, urban planning, construction, logistics, industry, agriculture and food systems, power grids, control and prevention of epidemics or disaster prediction (Botín-Sanabria et al., 2022; Tzachor et al., 2022b).

For instance, DTs can simulate and predict energy production by various energy sources including renewable energies or even consumption behavior. In doing so, they help to plan smart and stabile energy infrastructure or make personal recommendations for energy efficiency measures (Tzachor et al., 2022b). In urban planning, DTs can, among others, support noise, pollution and heat monitoring, improve traffic planning or waste and water management. DTs allow stress-testing and real-time responses to systemic shocks such as pandemics, wars or extreme weather events (Mehrabi, 2023). A broad application field for DTs is in the food and agricultural sector, whose transgression toward sustainability can be supported by digital innovations (Garske et al., 2021; Tzachor et al., 2022a; Heyl et al., 2023). With a view to the vulnerability of agricultural sites to external stressors such as climate warming, DT's ability to monitor and predict environmental changes seems to be a very valuable part of smart agriculture. DTs enable stakeholders in the food and agricultural sector to optimize resource and infrastructure use through monitoring and process evaluation, e.g., of environmental data, livestock emissions, inputs to crops such as Phosphorus and Nitrogen fertilizers, consumption data and supply chain tracking (Botín-Sanabria et al., 2022; Tzachor et al., 2022a,b; Purcell et al., 2023). Besides, DTs allow experimenting with nature-based solutions, e.g., for land management and flood reduction (Tzachor et al., 2022a; Ruangpan et al., 2023). In providing a virtual laboratory for what-if simulations, DTs empowers users to evaluate the state and predict the impact of intended or unintended alternations of real-world systems and their management, thus allowing for optimal mitigation or adaption strategies (Tzachor et al., 2022a; Purcell et al., 2023). This is possible not only for individual products, agricultural sites, cities, etc., but ultimately for the whole planet (Li et al., 2023).

**Figure 2:**
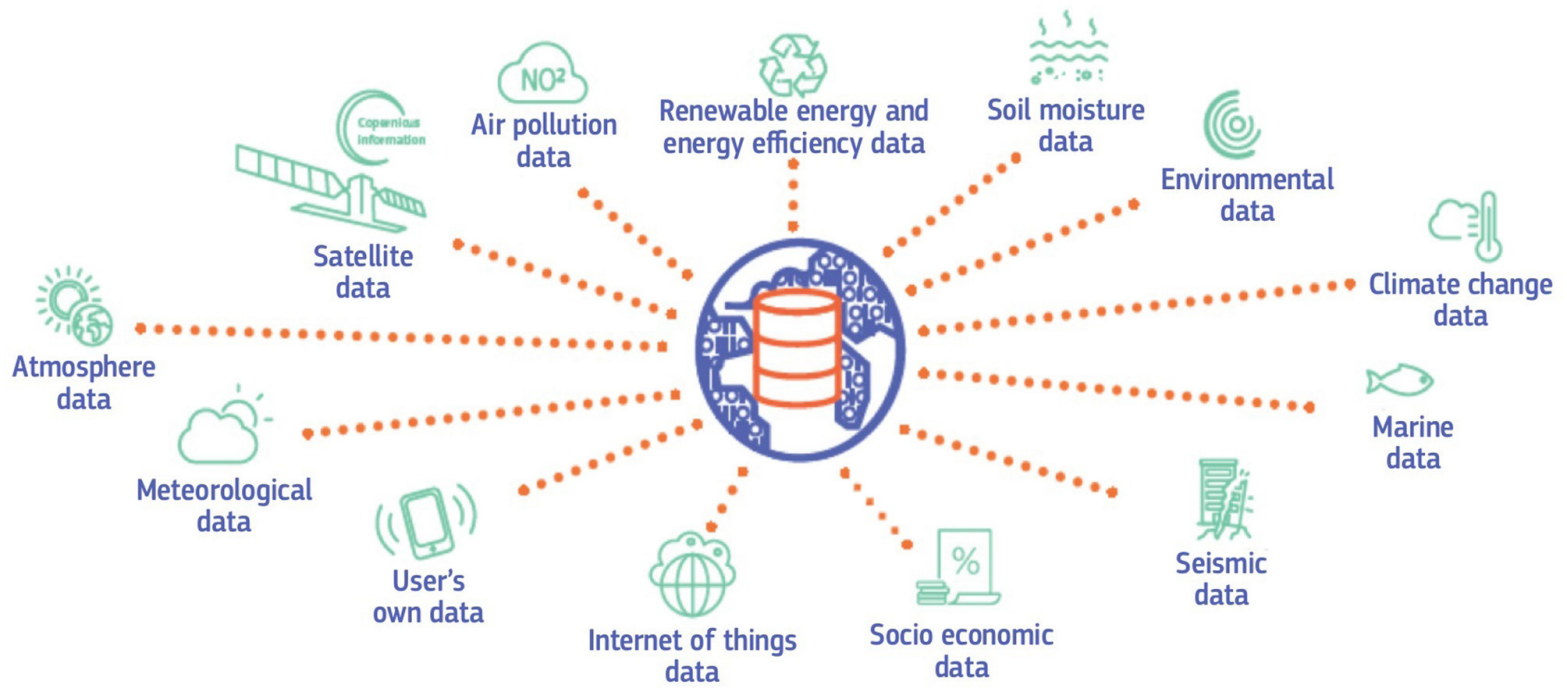
Data lake (data sources for DestinE) (European Commission, 2022b).

With Destination Earth (DestinE), the EU established the goal to generate a highly accurate, complete DT of the Earth by 2030 (Bauer et al., 2021; European Commission, 2022b; Li et al., 2023). Aiming at monitoring and predicting environmental change and human impact, DestinE shall support the EU's green transition toward climate neutrality by 2050 and to reach further environmental goals of the European Green Deal including fossil-free, circular production with zero waste and zero pollution (Bauer et al., 2021). The initiative seeks to develop a knowledge platform for multi-stakeholder collaboration, information sharing, and policy advice to enable the EU and Member States decision-makers at all levels to adapt policies to deal with ecological challenges regarding adaptation and mitigation (Botín-Sanabria et al., 2022; European Commission, 2022b). The Earth DT shall combine observations with an Earth system model and human subsystems such as water, food, energy and resource management to make predictions about their influences on each other (Bauer et al., 2021). For instance, the simulation of atmosphere, oceans, ice and land-cover with high precision, i.e., with a 1-km resolution in real-time, enables forecasts of floods, droughts and fires (Fissore et al., 2023; Li et al., 2023). In a first step, DTs for disaster forecasting and climate adaptation are created (European Commission, 2022b). In parallel, an open core service platform and a data lake are developed in the first 30 months implementation period in 2021–2024. While the core service platform encompasses open, cloud-based and secure decision-making tools, the data lake will provide storage and access to data. The initial digital services will serve users from the professional public sector. It is foreseen to expand the services to scientists, the private sector and the general public (European Commission, 2022b). Figure 2 provides an overview of the data sources for the data lake of DestinE, illustrating that vast amounts of natural and socio-economic information have to be collected and processed to build the Earth DT.

Like conventional simulation models, DTs can help to understand the drivers of change and identify options for future adaption to and mitigation of change (Bauer et al., 2021). However, in comparison, traditional simulation models use offline and a more static data basis (Bauer et al., 2021; Botín-Sanabria et al., 2022; Tzachor et al., 2022b). DTs in turn use AI and mathematical techniques to combine observation data and model simulations optimally to close gaps in the other: variables not observed everywhere all the time are filled by models and ML, while approximations for unknown and spatially unresolved processes of models are constrained by observations (Bauer et al., 2021; Tzachor et al., 2022b). The resulting data assimilation helps to depict evolving states of the represented system over time (Li et al., 2023). Hence, one advantage of DTs compared to model simulations is that they can assess and compensate model process errors and gaps through data assimilation. Further benefits of DTs over Earth system modeling include a much greater spatial and thereby physical realism and the ability to monitor and predict natural and human perturbations even on multi-decadal time scales. Besides, DTs are able to combine various sources of big data and optimize observation networks by evaluating their information content (Bauer et al., 2021; Li et al., 2023). Moreover, using a cloud-based infrastructure, DTs can give even non-expert users full access to data and data analytics toolkits (Bauer et al., 2021).

DTs should integrate the human dimension of the Earth system, i.e., impacts such das greenhouse gas emissions, land-use change, resource consumption and pollution (Bauer et al., 2021). They should enable scientists and policymakers to assess environmental change and human influence to make decisions supporting sustainable development within safe planetary boundaries (Li et al., 2023; Rockström et al., 2023).

General prerequisites for developing and implementing DTs successfully include sufficient computational and technological capacity including enabling technologies for DTs such as 5G, high performance computing for ML, big data assimilation, and cloud computing. Moreover, data availability, i.e., available, accessible and compatible data as well as standardized, interoperable tools for data processing and usage are necessary (Botín-Sanabria et al., 2022; Tzachor et al., 2022b). To this end, large-scale and high-frequency data collection, among others by sufficient sensor coverage, is required. At the same time, related to data, issues of trust, privacy, security, convergence and governance, acquisition and large-scale analysis arise (Botín-Sanabria et al., 2022). Furthermore, sufficient funding for DT projects is necessary, including for poorer nations which struggle to build and use expensive DTs due to lacking funds and underdeveloped digital infrastructure. In general, DT technology development und usage should be inclusive, i.e., accessible for people from various countries and sectors, neither excluding small enterprises nor minorities and overcoming the digital divide (Botín-Sanabria et al., 2022; Tzachor et al., 2022a,b; Mehrabi, 2023).

This article focusses on data governance as prerequisite for DT projects in the EU. It examines whether the political and legal provisions of the EU on data governance enable or hinder the successful implementation of DTs. The focus is on DTs which aim at supporting the sustainable transformation. Since environmental damage is – besides using fossil fuels – strongly connected to land-use and thus to agriculture and livestock farming in particular (IPBES, 2019; Ekardt, 2020; Weishaupt et al., 2020; Stubenrauch et al., 2021; IPCC, 2022), accessible and high-quality Earth observation data play an crucial role for the following analysis. The article is structured as follows: after a description of the methodology, the results of the legal analysis of the EU data governance are presented, starting with an overview of the EU Data Strategy (Chapter 3.1), followed by an examination of cross-sectoral (Chapter 3.2) and sector-specific legislation (Chapter 3.3). The last chapters discuss whether the existing EU strategies and legal acts on data governance promote the development of DTs for sustainable transformation in line with the EU Green Deal and international binding environmental targets on climate and biodiversity protection.

## 2 Materials and methods

This article applies a qualitative governance analysis (Ekardt, 2020; Weishaupt et al., 2020), also called legal impact analysis, to evaluate the impact of the EU's legal acts and policy strategies on data governance with relevance for DTs in the field of sustainable transition. The analysis seeks to complement the natural scientific and technical research on DTs by governance aspects. It aims at answering the following research questions: How can DTs support the transition toward sustainability? What are the success factors for DTs? In particular, how must data governance be designed to successfully implement DTs for sustainability? With its legal acts and strategies on data governance adopted in recent years, does the EU paves the way for DTs that promote sustainability?

To answer the first two research questions and to give an overview on DTs, the first step of our analysis was a literature review. The review encompassed Google Scholar and the portfolios of relevant scientific publishers and pertinent journals such as Springer, Frontiers, MDPI and Nature. Search terms included “digital twin,” “digital twin sustainability,” “digital twin agriculture,” “Destination Earth,” “data governance,” “EU data strategy,” “EU data governance act.” The focus of the review was on publications published from 2021 to 2023 and particularly DT publications with a focus on sustainability, SDGs and the EU Green Deal as well as on challenges related to data governance. However, none of the publications evaluated the EU's Data Governance. Furthermore, we used snowballing to identify further relevant publications. Besides, the EU Project DestinE was investigated using the relevant websites of the EU and the implementing organizations. The findings of the literature review are primarily included in the first chapter of the article.

The second step of the qualitative governance analysis encompassed the legal analysis, starting with the search in the data base for EU law eurlex and the website of the EU Commission to identify the relevant legal acts and strategies. These acts and strategies were assessed with regard to their potential to promote DTs for sustainability, keeping in mind the prerequisites for implementing and applying DTs. The legal analysis includes the interpretation of legal norms with the usual legal methods of grammatical, systematic, teleological and historical interpretation.

The qualitative governance analysis measures the effectiveness of legal instruments against the internationally binding environmental targets that provide the framework for any legal and policy initiative. Hence, DTs, which are supposed to support sustainability, shall never counteract the goal of the Paris Agreement and the Convention on Biological Diversity. Both environmental targets have a basis in human rights that also include a precautionary dimension and the polluter-pays principle (Ekardt, 2020; Ekardt et al., 2022). Moreover, when assessing the effectiveness of legal acts and policy instruments, findings from behavioral studies which shed light on typical governance problems such as shifting and rebound effects, enforcement and depicting problems, and lacking target stringency were taken into account (Paul et al., 2019; Ekardt, 2020; Kanter et al., 2020; Ekardt et al., 2022). The findings of the governance analysis are presented in Chapter 3 and critically discussed in Chapter 4.

## 3 Results–legal analysis of the EU data governance

Every day, large amounts of data related to Earth systems, i.e., big Earth data, are gathered (European Commission, 2020b; Li et al., 2023). These large-volume datasets have to be processed, organized and governed (Li et al., 2023). Big Earth data has a wide range of data sources including remote sensing by satellites and drones and ground-based sensors, *in situ* and laboratory analysis including field experiments and surveys, data collected by smartphones and the Internet of Things, simulation and re-analysis and social sensing, i.e., diverse data related to human activities, behavior and population (Li et al., 2023). Data from all these categories provide the basis for representing the Earth system or its subsystems in a DT (Li et al., 2023).

**Figure 3:**
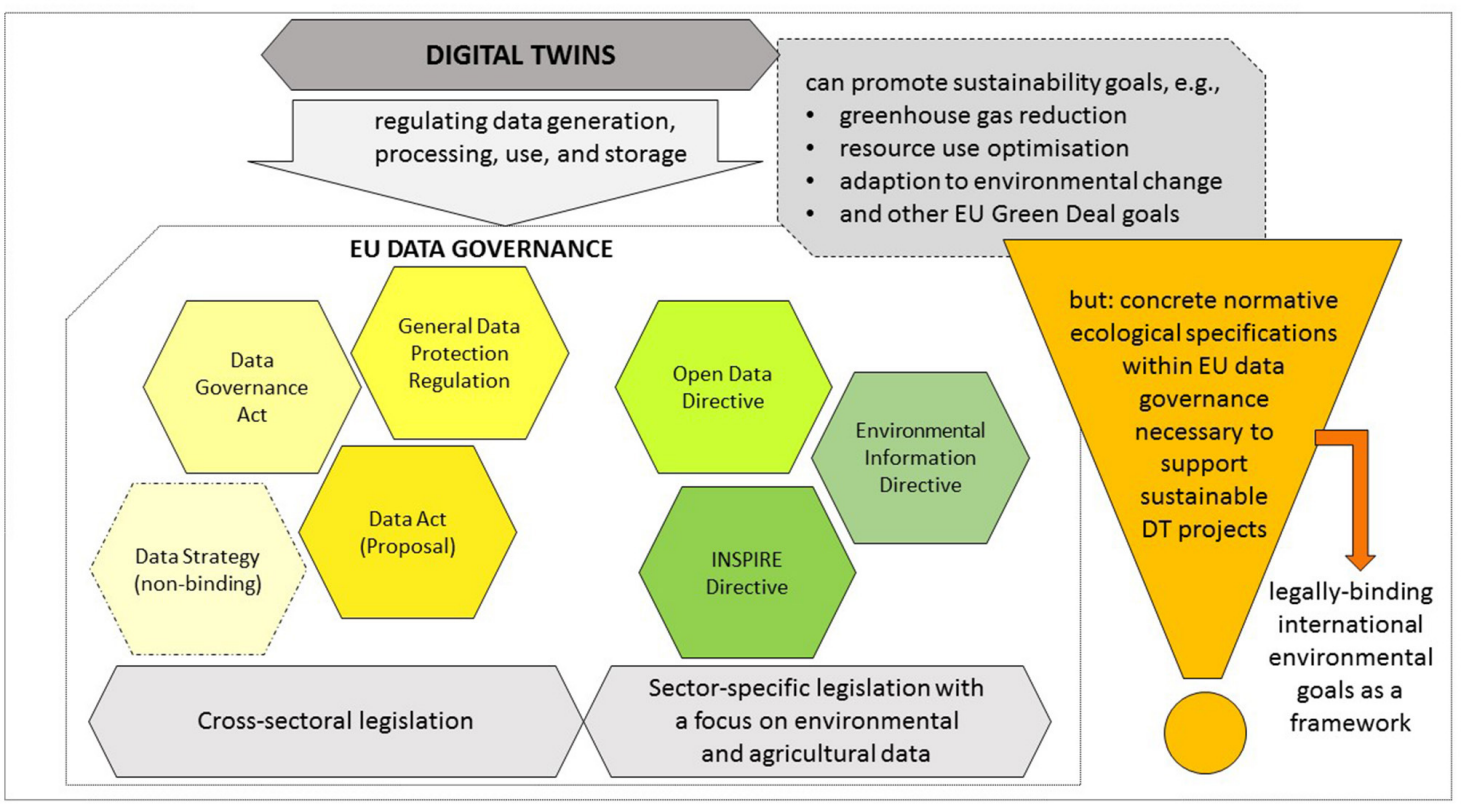
Digital twins, EU data governance and sustainability goals (own figure).

Yet, the advantages of big data processing and digital innovations including DTs can only be exploited when an appropriate data governance is in place, providing for effective, open and fair (see below) data use and collaboration (Li et al., 2023; Purcell et al., 2023). From a legal perspective, the use and deployment of DTs raises questions about the generation, use, processing, and storage of data. The first legal question concerns the data sources for DTs, in particular whether the data is obtained purely from internal or external data sets. In the legally more challenging case of external data mining, trustworthy data spaces that facilitate data access as well as leeway for individual agreements are required. For DT applications which affect the source code of a software, appropriate rights of use must be granted. If, in addition, data sets are processed that allow conclusions to be drawn about natural persons, a conflict with the provisions of data protection legislation arises, calling for an adequate normative and technical response. Once a DT has already been implemented and third parties are supposed to use it, the question arises as to what extent the DT can be protected against duplication, unlawful reading and disclosure of relevant information. While the exchange of data between the data owner and the producer of a DT is regulated by the General Data Protection Regulation (EU) 2016/679 (GDPR) (European Union, 2016) and further EU legislation, the transfer and reproduction of code primary concerns the areas of product liability, copyright and tort law, for which the national legislator must establish suitable mechanisms. In the following, the national legislation framework will be left aside and attention will be focused on the level of Union law. Given the project's ambition to bring together data from various public and private sources to develop an Earth DT, cross-sector and sector-specific regulation become relevant – with tensions arising between data access/data use and data privacy. How all this is regulated within the framework of the EU data governance is examined in the following. Figure 3 shows the most relevant EU legal acts for DT projects in the area of environment and agriculture and highlights the need of concrete ecological normative specifications when developing DTs that should promote sustainability.

### 3.1 The EU data strategy

The EU data governance, consisting of a bundle of data-related legal acts and policy strategies, aims at creating a single European data market which ensures availability and flow of data, investments in standards, tools, infrastructures and competences for handling data.

According to the EU Data Strategy from 2020, data collection and use shall comply with the European rules and values such as personal data protection, consumer protection and competition law – and be consistent with the EU's goals of the Green Deal (European Commission, 2020b). At the same time, the potential of using large amounts of data shall be exploited and should be made available to all instead of being owned by a small number of big tech firms or being accessible only for government authorities (European Commission, 2020b). One main principle of the EU's data governance is the FAIR-Principle, meaning that data needs to be findable, accessible, interoperable and reusable (European Commission, 2020b).

Before this background, the EU has taken various steps to accelerate the digital transformation in the EU. Among them is the EU Data Strategy from 2020 (European Commission, 2020b), which is also part of the Commissions' Communication on “Shaping Europe's digital future” (European Commission, 2020c) and a White Paper on AI (European Commission, 2020a). The Strategy outlines policy measures and funding opportunities for the EU data economy and points the way for future legal acts on data governance (European Commission, 2020b).

In addition, one priority of the Data Strategy is to operationalise a legislative framework for the governance of common European data spaces for strategic sectors such as mobility, health, energy, agriculture, industry and finances (European Commission, 2020b, 2022c). The spaces, which will make data available on a voluntary basis, shall ensure availability, quality and interoperability of data. They shall include data-sharing tools and platforms as well as enabling data governance frameworks (European Commission, 2020b, 2022c). The design of the data spaces has to comply with European rules and values. Access and use of data shall take place in a secure, clear, fair, transparent, proportionate and non-discriminatory manner (European Commission, 2022c). The spaces shall be interconnected with the European Open Science Cloud (EOSC) and the Copernicus services (European Commission, 2020b). The latter are important services for making freely available data related to atmosphere, marine, land, climate change, security and emergency in the EU. They are managed through access points called Data and Information Access Services (DIAS) and the Copernicus Reference Data Access dashboard (CORDA). Further environmental data, e.g., on hydrography, elevation, land cover, natural hazards and fire are available through the INSPIRE Geoportal. Also, the European Environmental Agency provides free environmental data and services in its geoportals (Fissore et al., 2023). Besides, the land-use database Open Land Use (OLU) can support DT projects such as DestinE in developing a high-precision digital model of the Earth by providing land-use and land-cover data (Kepka et al., 2022).

The Green Deal data space is one data space envisaged by the European Data Strategy (European Commission, 2020b, 2022c; Kepka et al., 2022). It encompasses DestinE and “GreenData4All,” an initiative that includes, among others, the evaluation of the INSPIRE Directive (European Union, 2007) and the Access to Environmental Information Directive (European Union, 2003; European Commission, 2020b, 2022c) (see Chapters 3.3.2 and 3.3.3). Moreover, the Green Deal data space is strongly connected to further data spaces such as the data space for agriculture. While the Green Deal data space aims at supporting actions on climate change, circular economy, zero-pollution and biodiversity, the agriculture data space shall enhance sustainability of the agricultural sector. Yet, sustainability in the agricultural sector makes compliance with the Green Deal goals necessary. Thus, both data spaces show strong interlinkages and can hardly be developed and implemented separately from each other. This is also true for all other planned data spaces, in particular those for energy, mobility or health.

Creating data spaces to ensure cross-border data use and re-use as well as further ideas and plans presented in the European Data Strategy are promising. However, their success depends on the design of legally binding acts which ensure, e.g., the implementation of FAIR principles and the success of data-driven projects. The relevant legal acts adopted to date include the General Data Protection Regulation (GDPR) (European Union, 2016) and the Data Governance Act (DGA) (European Union, 2022a). Supplementing the DGA, the Commission proposed a Data Act (DA) (European Commission, 2022a). In addition, sector-specific legislation on data exists. For the present topic, the Open Data Directive (ODD) (European Union, 2019a) and the INSPIRE Directive (European Union, 2007) as well as the Environmental Information Directive (EID) (European Union, 2003) are relevant. Further regulations encompassed by the EU Data Strategy include the Regulation on the free flow of non-personal data (European Union, 2018) and the Cybersecurity Act (European Union, 2019b). Moreover, in the rapidly developing field of digital law, many other legal acts have been adopted or proposed in recent years, including the Digital Markets Act (European Union, 2022b), the Digital Services Act (European Union, 2022c) and the draft for an AI Act (European Commission, 2021). All these acts support the development of the single market for data from various legal perspectives such as competition law.

Below, we analyse the most important legal acts for realizing DTs, starting with the DGA and the proposed DA as cross-sectoral legislation including their relations to the GDPR. Examinations on the relevant sector-specific legislation follow.

### 3.2 Cross-sectoral legislation

#### 3.2.1 EU data governance act

##### 3.2.1.1 Objective

As the first element for implementing a single European data market and for establishing the necessary data infrastructure for a DT, the Regulation (EU) 2022/868, also known as Data Governance Act (European Union, 2022a), entered into force in June 2022. The horizontal regulation is binding in its entirety and directly applicable in all Member States by the end of September 2023 [Art. 38 DGA and Art. 288 para. 2 TFEU (European Union, 2009)]. The primary purpose of the DGA is to increase trust in data intermediaries, i.e., organizers of data sharing such as data marketplaces, and thus increase the availability of data (recitals 5, 32 DGA). The current reluctance of companies to use data intermediation services and share their data with other companies is, in the Commission's view, primarily caused by trust deficits in data markets (European Commission, 2020d). By helping to establish the necessary trust in data intermediaries, the DGA supports these intermediaries in gaining a larger user base. This aims to support a flourishing data exchange in the EU (European Commission, 2020d). In addition, the DGA is intended to prevent potentially anti-competitive behavior of data intermediation service providers such as self-preferences or unequal treatment (recital 33 DGA) (European Commission, 2020d).

##### 3.2.1.2 Subject of regulation–data altruism and data intermediaries

The DGA is intended to facilitate the enhanced re-use of data in the possession of public bodies that are protected by other rights. These special protection rights include commercial and statistical confidentiality, the protection of intellectual property of third parties and the protection of personal data (Art. 3 para. 1 DGA). The EU legislator stipulates certain requirements for the further use of personal data. Such data must either be anonymised, modified or made accessible only within a secure processing environment as defined in Art. 2 No. 20 DGA (Art. 5 para. 3 DGA). Any conditions for re-use must be non-discriminatory, transparent, proportionate and objectively justified (Art. 5 para. 2 DGA). However, the DGA does not oblige public sector bodies to allow the re-use of data (Art. 1 para. 2 DGA). In particular, it does not grant users a right to access data.

To help users find their way through the “data and information jungle,” the DGA demands Member States to establish central information points that provide easy access to relevant information. Furthermore, Member States are required to designate one or more competent bodies to assist public authorities in fulfilling their new responsibilities (Art. 7 DGA). For instance, the public sector bodies should receive support in ensuring data processing that preserves privacy, confidentiality, integrity and accessibility of data.

The core concern of the DGA is to ensure that data is made available quickly. The public sector bodies (if applicable also the competent bodies according to Art. 7 DGA) must therefore decide on a request for further data use within 2 months (Art. 9 para. 1 DGA). The DGA also aims at supporting data altruism, i.e., “the voluntary sharing of data on the basis of the consent of data subjects to process personal data pertaining to them, or permissions of data holders to allow the use of their non-personal data without seeking or receiving a reward […] for objectives of general interest as provided for in national law” (Art. 2 No. 16 DGA). The legal definition gives examples of the public purposes which include healthcare, combating climate change, improving mobility, public policy making or scientific research purposes in the general interest.

In addition to the provisions of Art. 5, the DGA mainly establishes basic organizational conditions: these include, for example, regulations for a public register on recognized data altruistic organizations (Art. 17 et seq. DGA), transparency requirements (Art. 20 DGA), specific requirements to safeguard rights and interests of data subjects and data holders (Art. 21 DGA), provisions for a so-called rulebook (Art. 22 DGA) as well as regulations for monitoring by the competent authorities (Art. 23 et seq. DGA). Finally, Art. 25 DGA contains requirements for a “European data altruism consent form.” The EU legislator hopes that the standardized form will make it easier to collect data on the basis of data altruism (Art. 25 para. 1 DGA). To allow its use in certain sectors and for different purposes, the consent form is intended to be modular (Art. 25 para. 2 DGA). The Commission is empowered to adopt implementing acts specifying the details.

Moreover, the DGA establishes a notification and monitoring framework for so-called data intermediation service providers (Art. 2 No. 11 DGA). These aim to establish business relationships to share data between an undetermined number of data subjects or data owners (Art. 2 No. 7, 8 DGA) on the one hand and data users (Art. 2 No. 9 DGA) on the other. Data intermediation service providers are generally obliged to be registered by the competent authority of the Member State (Art. 11 para. 1 DGA). In addition, anyone offering data intermediation services must fulfill several conditions (Art. 12 DGA), in particular the principle of purpose limitation: the provider is not allowed to use the data for which it provides services for purposes other than to put them at the disposal for data users (Art. 12 lit. a DGA). Changes of purpose are thus excluded in principle. Metadata may only be used to further develop the service (Art. 12 lit. c DGA). These conditions express the special neutrality responsibility of the data intermediation service provider (Spindler, 2021). The intermediary is also obliged to provide for appropriate technical, legal and organizational measures to prevent the unlawful transfer of or access to non-personal data (Art. 12 lit. j DGA). In addition, the provider must ensure an adequate level of security for the storage, processing and transmission of non-personal data and especially of sensitive information (Art. 12 lit. l DGA).

Monitoring compliance with the requirements on data intermediary services according to Chapter III (Art. 10-14) of the DGA ex post is the responsibility of the competent authority (Art. 14 para. 1 DGA) (Spindler, 2021). If the authority finds an infringement, it can demand within a reasonable period of time (or immediately in the case of serious violations) that the provider ceases the infringement. In addition, the authority can, for example, impose dissuasive financial penalties (Art. 14 para. 4 lit. a DGA).

Besides, the DGA calls for establishing a European Data Innovation Board (EDIB) as an advisory and support unit. The Board is an expert group composed of, among others, representatives of certain Member State authorities, the European Data Protection Board (EDPB), the European Data Protection Supervisor (EDPS), the European Union Agency for Cybersecruity (ENISA) and the European Commission (Art. 29 para. 1 DGA). In particular, the EDIB shall advise and assist the Commission in developing a consistent practice of data altruism across the EU. In addition, the EDIB shall propose guidelines for the Common European data space (Art. 30 DGA).

##### 3.2.1.3 Relation to the GDPR

The DGA does not affect the scope of the GDPR, so that both regulatory provisions apply in parallel (European Union, 2022a; Specht-Riemenschneider, 2022). However, given the fact that certain elements of the DGA affect personal data, the measures of the DGA have been designed to fully comply with data protection rules and to strengthen the control of natural persons over the data they generate. As far as the re-use of public sector data is concerned, the fundamental rights to data protection, privacy and property (ownership rights to certain data containing, e.g., trade secrets or intellectual property rights) are respected (European Union, 2022a). Data intermediaries must also comply with the existing data protection regulations. Hence, the provisions of the DGA are in line with the existing rules on data protection of the EU (European Union, 2022a).

#### 3.2.2 EU data act proposal

##### 3.2.2.1 Objective

In February 2022, the Commission presented a proposal for a Regulation on harmonized rules on fair access to and use of data, also known as the Data Act. It is a key pillar of the European Data Strategy. The DA is intended to supplement the provisions of the DGA on the basis of Article 114 TFEU in order to create a single market for data that grants consumers more rights, strengthens the negotiating position of smaller companies and de-monopolizes data (European Commission, 2020b; Specht-Riemenschneider, 2022). On the normative foundation of the DGA, the DA specifies the preconditions for specific data use or value creation. Another aim of the DA is to strike a balance between the needs of the digital economy and a fair and secure data use. Both aspects are important in order to implement innovations such as DTs (European Commission, 2022a).

##### 3.2.2.2 Subject of regulation–interoperability and “access by design”

Like the DGA, the proposed DA is based on a horizontal approach (Art. 1 DA Proposal) (Specht-Riemenschneider, 2022). Existing sectoral rights and obligations on data access and use must not be changed. However, subsequent legal acts have to be oriented toward the future DA and are therefore to be brought into line with its provisions. At the same time, the EU Commission emphasizes that the DA leaves enough scope to adopt detailed regulations if required by sector-specific regulatory objectives (European Commission, 2022a).

Chapters I–IV of the draft regulation focus on rules for governing data access between data owners, users and third parties as data recipients. Chapter V specifies the access rights of public authorities. Chapters VI, VII, IIX DA Proposal include regulations on the portability of data between processing services and guarantees the protection of non-personal data against unlawful transfers to or state access from third countries. Besides, interoperability requirements for data are stipulated. The regulations are highly relevant for the functioning of the single data market. In particular, in order to promote the European data market and reduce existing hurdles, the DA intends to create a legally secure framework for data transfers. It seeks to cushion the effects of asymmetric market positions: small and medium-sized enterprises (SMEs) are to be protected by the development and provision of – optionally usable – fair model contractual terms and conditions in the value-creating transfer of data (Art. 34 DA Proposal).

The draft DA grants public bodies the right to privileged access to data if they can prove an “exceptional need,” e.g., due to a natural disaster (Art. 14 para. 1 DA Proposal). This may concern not only company data, but also personal data. If necessary, this data must be pseudonymised in accordance with Art. 4 No. 5 GDPR before government agencies receive them (European Commission, 2022a).

In order to facilitate switching between data processing services and to counteract dominant market positions of individual companies, in particular to prevent so-called “lock-in effects,” the draft DA stipulates interoperability requirements (Art. 28 DA Proposal). This is consistent with the protection of the individual data user by access rights to data: the data holder should make the data available to the user of a networked product or related service without delay, free of charge and, where appropriate, continuously and in real time (Art. 4 para. 1 DA Proposal). Moreover, the idea of Art. 3 para. 1 DA Proposal is “access by design”: products are to be manufactured in a way that the data generated during their use is easily and securely accessible. For projects that rely on data, such as DTs, this regulation is quite beneficial.

##### 3.2.2.3 Relation to the GDPR

The application scope of the proposed DA includes personal and non-personal data. Thus, the protection of privacy may be affected by the DA. In principle, Art. 1 No. 3 and the recitals of the DA Proposal state that the Regulation merely complements Union law on data protection and privacy, in particular the GDPR and the Directive on privacy and electronic communications 2002/58/EC (European Union, 2002; European Commission, 2022a). No provision of the DA shall be applied or interpreted in a way that weakens or restricts the right to protection of personal data or the right to privacy and confidentiality of communications. Hence, the DA is no lex specialis to the GDPR (Specht-Riemenschneider, 2022). As a result, in cases in which the DA applies to personal data, provisions of the DA and the GDPR must be complied with. In order to mitigate the risk that the application of existing data protection rules could be affected or undermined by an interpretation or implementation of the DA, the EDSA and the EDPS call on the legislator to strengthen the wording of Art. 1 para. 3 DA Proposal. They propose to explicitly state that, as far as personal data is concerned, data protection rules shall prevail over the provisions of the DA in case of conflict (EDPS and EDPB, 2022; Specht-Riemenschneider, 2022). Yet, in general, the proposed DA is designed in a way that avoids conflicts of data access claims of data users, third parties and data subjects (Specht-Riemenschneider, 2022). One striking difference between the regulations, however, is that according to Art. 4 and Art. 5 DA Proposal, the user now has a right to data being made available to him or to third parties continuously and in real time instead of only once upon request. This provision could accelerate data use for DT projects.

### 3.3 Sector-specific legislation

#### 3.3.1 Open data directive

As part of the European Strategy for Data, the Directive (EU) 2019/1024 on open data and the re-use of public sector information (Open Data Directive) (European Union, 2019a) applies to government-held data (public sector information) (European Union, 2019a; European Commission, 2020b). The European legislator has revised the ODD in 2019 and adapted it to the data use requirements of key technologies such as AI, which are also relevant for the development DTs. Since 2003, the goal of the ODD is to promote data-based business models on the basis of publicly financed data. The regulatory content can basically be divided into two dimensions: on the one hand, the ODD aims at preventing distortions of competition in the internal market through public authorities with regard to value-added services that are developed and offered based on public sector data. This purpose is pursued by the principle of non-discrimination and the prohibition of exclusive arrangements (Art. 11, 12 ODD). On the other hand, the Directive harmonizes conditions for the re-use of accessible data regarding, e.g., formats and charges (Art. 5, 6 ODD). With the revised version, the EU legislator has extended the application scope beyond public bodies to include public undertakings in specific areas of services of general interest. Furthermore, research data is now covered. In detail, publicly funded research data shall be openly available by default and their access shall be compatible with the FAIR principles (Art. 10 ODD) (see above).

The new ODD sharpens the principles of limiting charges on data re-use and introduces special requirements for dynamic data. In the future, data that is regularly updated, such as sensor data, should be available in real time via application programming interfaces (APIs) (Art. 5 ODD). In addition, so-called high-value data sets are introduced, which are to be specified in thematic categories by implementing acts. In Art. 13 para. 1 in conjunction with Annex I ODD, the Directive defines broad thematic categories (geospatial, earth observation and environment, metrology, statistics, enterprise and mobility), whose legal scope could potentially cover most data types. The specific data covered by the abstract legal wording of “high-quality data sets” is determined by the Commission through delegated acts (Art. 13, 14 ODD). High-quality data sets are considered to be of particular importance for society, the environment and the economy. Public bodies and companies providing services of general interest are required to make such data available not only free of charge, but also in machine-readable formats through suitable programming interfaces and, where appropriate, as a mass download free of charge. These data requirements may well benefit DT projects.

#### 3.3.2 INSPIRE

For the creation of an Earth DT, as envisaged in DestinE, spatial environmental data from the Member States are invaluable. These data can be used to analyse, derive and assess environmentally relevant parameters, such as the distribution of agricultural land or natural hazard risks. To improve the availability, quality, organization, accessibility, and shared use of spatial data, the legal, organizational, and technical basis for a pan-European spatial data infrastructure was created in 2007 with the Directive 2007/2/EC establishing an Infrastructure for Spatial Information in the European Community (INSPIRE Directive) (European Union, 2007). For the necessary integration of national infrastructures, the Member States were obliged to provide access to information on spatial data or spatial data sets via a geoportal managed by the Commission and via self-established access points. Additionally, requirements are placed on the properties and contents of the spatial data sets, i.e., they must be available in electronic form and relate to one or more of the topics listed in Annexes I-III of the Directive (Art 4 para. 1 INSPIRE Directive). Spatial data services are used to process the spatial data and the associated metadata. The services shall be easy to use, available to the public and accessible via the Internet or other appropriate means of telecommunication (Art. 11 INSPIRE). However, access to such spatial data sets is not granted unlimitedly. Access may be restricted or require a consent of the data holder, if it would adversely affect, e.g., international relations, public security or national defense or the confidentiality of personal data, commercial or industrial information, provided that such confidentiality is granted under national or Union law (Art. 4, 13 INSPIRE Directive). However, the public interest in disclosure shall be weighed against the interest in imposing restrictions or conditions on access in each individual case (Art. 13 para. 2 No. 2 INSPIRE Directive).

As mentioned above, the INSPIRE Directive as well as the Environmental Information Directive (see below) are revised within the GreenData4All initiative. Both directives will be modernized and adapted to the current state of the art, so that data necessary for ensuring compliance with the EU environmental legislation can be collected, processed, analyzed, and shared on a large scale (European Commission, 2020b, 2022c). It remains to be seen whether the revision can solve, for example, existing licensing problems of the INSPIRE Directive. Currently, the INSPIRE licensing agreement for open publication of spatial data cannot be used for all situations where vast, multi-temporal, multi-scale and multi-platform datasets are combined due to single restrictions (Kepka et al., 2022). Besides, neither all data nor all data licenses are compatible, which makes data combination complicated even in cases of open data, e.g., when it is forbidden to alter or add data (Kepka et al., 2022). Yet, using data for DestinE and other DT projects by various users, makes openly published data without major restrictions necessary (Kepka et al., 2022).

#### 3.3.3 Environmental information directive

Access rights to environmental information have a long tradition. They date back to the Aarhus Convention (UNECE, 1998) of 1998 and were comprehensively broadened under the Directive 2003/4/EC on public access to environmental information (Environmental Information Directive) (European Union, 2003). The Directive stipulates comprehensive obligations to provide all information that may be relevant to environmental protection and an almost unrestricted access right upon request (Art. 2 No 1 lit. a-e EID). Requested environmental information must be made available as soon as possible (Art. 3 para. 2 EID). Besides, officials are required to support the public in seeking access to information. Public authorities have to inform the public adequately of the access rights they enjoy (Art. 3 para. 5 EID). Finally, in cases in which the requested information is not available to the authority, authorities are obliged either to forward the request as quickly as possible or to inform the applicant of the authority to which, in their opinion, he can apply for this information (Art. 4 para. 1 lit. a EID). The extensive right to data access for everyone may only be restricted for reasons of ensuring effective public authority action, or if the disclosure of the requested information negatively impacts legal interests such as trade secrets, the protection of intellectual property, the confidentiality of personal data or the protection of the environment, e.g., regarding the location of rare species (Art 4 para. 2 EID). The principle “as open as possible, as closed as necessary” is also enshrined in the EU Framework Programme for Research and Innovation Horizon Europe (European Union, 2021). With respect to research data, Regulation (EU) 2021/695 establishing Horizon Europe [Horizon Europe Regulation (European Union, 2021)] once again anchors that research data should be findable (openly) accessibly, interoperable and reusable according to the FAIR principles (Art. 14 Horizon Europe Regulation). These principles have the potential to support the development and application of DTs in various fields including those connected to sustainable development.

## 4 Discussion

The EU's strategies and legal acts on data governance set the course for transparent structures and for making data accessible, thus supporting DT projects such as DestinE which aim at stopping environmental degradation and promoting sustainable transition. In addition, some cross-sectoral provisions such as Art. 5 II DGA, build a link between data use and the dissemination of “European values.” Such a value link can also be found in the European Data Strategy, in which open data access is positively linked to sustainability goals. However, the provisions fail to show why and how the creation of a single European data market and open and fair data use should automatically reduce environmental pressure. At first glance, the logic behind this seems plausible: the more data is used, e.g., for DTs, the more societal value can be derived from it. However, environmental protection and the development of sustainable products and services do not primarily depend on the existence of a functioning data space. Rather, the legal framework that applies for the data space is decisive. Yet, neither the DGA nor the DA Proposal are sufficient to guarantee an effective link between data governance and sustainability goals.

The sector-specific regulation, i.e., the INSPIRE Directive, the ODD and the EID, are comparatively more suitable to promote sustainability objectives within the framework of digitalisation. Taking into account that environmental change does not stop at borders, the INSPIRE Directive creates an EU-wide infrastructure for spatial information including cross-border use and interoperability of data, thus supporting the development of EU environmental legislation. Besides, the extended scope of the ODD to research data improves knowledge dissemination and reduces knowledge dependencies. In addition, the access rights to environmental information guaranteed by the EID create a culture of participation (Stuermer et al., 2017).

In fact, much of the data needed for developing and implementing DTs already exists. For instance, crop production is monitored by satellites; traffic data for major roads, railways, waterways and ports are available due to GPS; production, consumption and trade data exist for many commodities and regions (Mehrabi, 2023). Putting together all these pieces and closing knowledge gaps in predicting the future, DTs provide a chance for informed, data-driven decision-making and thereby supporting sustainable development (Bauer et al., 2021; Li et al., 2023; Mehrabi, 2023). In particular, a good database can help to derive concrete, effective political measures, e.g., for climate protection. However, a DT cannot decide whether measures to protect the climate and other environmental goods are necessary at all. Neither can the start of ambitious measures to tackle the pressing ecological and societal challenges of the twenty first century wait for more data to become available or processed. Hence, collecting, using and sharing data are only single steps for environmental policy which need to be complemented by enacting effective policies.

Moreover, several scientific organizations have been presenting data and scenarios on environmental change for a long time, e.g., the Intergovernmental Panel on Climate Change (IPCC) on climate change. Yet, these studies did not trigger the necessary rapid transition to sustainability. Likewise, Earth system models, e.g., on land use or climate trends, have existed for a long time. However, their precision and completeness are often overestimated and they are mistaken as predictions or even as normative statements (Wieding et al., 2020). While DTs offer advances on spatial precision and real-time data availability, they will not automatically stimulate the necessary shift in environmental and climate protection – especially since factual knowledge is not the major motivational factor for sustainable transition and behavior anyway (Ekardt, 2020; Heyl and Ekardt, 2022).

In contrast, proposals such as “testing climate targets virtually” (Li et al., 2023) cannot fall within the scope of DTs, since these targets are legally binding under international law and ambitious climate protection is also required under human rights law (Ekardt et al., 2022). Rather, DTs must incorporate the mandatory environmental goals of international, regional and national law. Similarly, suggestions that DTs offer a virtual space for newly developed technologies that may cause unintended harm und thus may be inhibited by the precautionary principle (Tzachor et al., 2022b; Li et al., 2023), must be taken with a pinch of salt. In virtual space, of course, experimentation is allowed. Yet, a direct transfer to the real world is not permissible, since no absolutely reliable DT of the Earth will ever exist.

DTs can only be an approximation to a real-world object, and every prediction of the future development bears uncertainties (Tzachor et al., 2022a). Although data on the Earth system is large, some data-related challenges such as quantifying ecological and social tipping points remain (Li et al., 2023). Since the Earth system is open, complex, non-linear and chaotic, ML methods and in particular deep learning can help to gain insights into these systems. However, despite methodological progress, limitations regarding interpretability and transferability are likely to remain, at least in the short-term (Li et al., 2023). Thus, DTs can fail to represent hidden biophysical feedback processes (Purcell et al., 2023). And although social sensing data are evolving and bring new opportunities to capture and quantify human dynamics, they are connected to high uncertainties (Li et al., 2023). Such uncertainty impedes decision-making, for example with regard to identifying sustainable development paths.

Moreover, as it is true for all AI applications, DTs and their predictions are only as good as their design and inputs allow them to be. In fact, the design of DTs might incorporate biases such as racism, sexism or misrepresentation of minorities. Design weaknesses may be further aggravated by combining multiple and co-depended ML models (Tzachor et al., 2022b; Purcell et al., 2023). Thus, again, informed decision-making is hindered and discrimination, exclusion and the digital divide may be fostered (Tzachor et al., 2022a,b). So far, the EU does not propose a sufficient remedy to overcome such difficulties.

Besides, similarly to other big technological innovations, DTs may potentially accelerate market centralization, external dependencies and wealth inequality, e.g., when small holdings cannot afford high investment costs for new technologies which provide competitors with a competitive advantage (Purcell et al., 2023). This makes a legal framework necessary which counteracts the concentration of power by individual tech companies and helps to overcome exclusion. The EU has laid the foundation for such a framework with the legal acts adopted or proposed as part of the data strategy, in particular with the DA Proposal.

In general, DT projects such as DestinE are more likely to succeed if the wide range of data sources and new technologies is made openly available (Kepka et al., 2022). A set of open-source gold standards to support open and equitable DT development in line with sustainability goals should be further developed and applied (Purcell et al., 2023). This also helps to ensure that public funding for DT projects is used efficiently and that data does not, e.g., have to be collected twice. Specifically, combining data for DT projects bears a chance to harmonize many mapping and modeling initiatives for various ecosystems (Fissore et al., 2023). Data-related standards should encompass data acquisition and processing, data representation, type of information including meta data and conditions under which different information can be used (Fissore et al., 2023). In addition, data quality, i.e., completeness and accuracy (Kepka et al., 2022), shall be ensured. High quality data needs to be available for the public good (European Commission, 2020b). Again, the EU data governance provides important starting points, particularly with regard to data altruism as stipulated in the DGA and the FAIR principles as laid down, i.e., in the ODD.

Moreover, the EU linked the legal framework on promoting data availability to data protection. Nevertheless, the EU legislator must continue to ensure data protection by using the latest technologies, e.g., regarding cryptographic tools, and to reduce security risks due to hacking, e.g., with a view to transport infrastructure, food or energy system fragilities (Mehrabi, 2023).

## 5 Conclusion

DTs are one example of many digital innovations that promise to promote sustainability. For instance, in the agricultural sector, which is strongly affected by environmental change such as extreme weather events on the one hand and is a major driver of environmental degradation on the other, DTs offer many opportunities for optimized resource management. But also with a view to further applications in other sectors, the goals of DTs and of sustainability show many interlinkages, e.g., regarding greenhouse gas reduction, resource-use optimisation or adaption to environmental change (Purcell et al., 2023). In line with that, the EU highlights the strong connection between sustainable and digital transformation in the context of the Earth DT (European Commission, 2022b).

If, as demanded by the EU itself, DTs should contribute to the implementation of the SDGs and the Green Deal (European Commission, 2020b; Bauer et al., 2021; Botín-Sanabria et al., 2022), internationally binding objectives on climate and biodiversity from the Paris Agreement of the Convention on Biological Diversity have to provide the framework for the development and application of DTs. However, due to the lack of concrete normative ecological specifications within the EU provisions on data governance, no sufficient regulatory responsibility for environmental protection was taken. Instead it remains with the vague hope for responsible action on the part of the actors, especially the companies, to promote sustainability by their DT projects. Thus, the EU still has some catching up to do in order to better support the development and application of DTs that promote sustainable transition.

Moreover, not only the application scope of DTs should consequently be linked to sustainability goals. DTs themselves should also be designed as sustainably as possible. This includes especially energy and resource consumption of a high-performance computational infrastructure, big data processing as well as expansive earth observation. The ICT sector is estimated to contribute 5% to 9% of the world's total electricity use and more than 2% of all emissions (European Commission, 2020b). Hence, in addition to covering energy consumption from renewable energy sources, energy consumption for this sector as a whole must be reduced as far as possible (Li et al., 2023). Besides, technology production should be oriented toward the principles of circular economy, zero waste and zero pollution.

## Data availability statement

The original contributions presented in the study are included in the article/supplementary material, further inquiries can be directed to the corresponding author.

## Author contributions

BG: Conceptualization, Formal analysis, Investigation, Methodology, Supervision, Visualization, Writing—original draft, Writing—review & editing. WH: Formal analysis, Writing—original draft. FE: Supervision, Writing—review & editing.
